# Genome-Wide Characterization of IQD Family Proteins in Apple and Functional Analysis of the Microtubule-Regulating Abilities of MdIQD17 and MdIQD28 under Cold Stress

**DOI:** 10.3390/plants13172532

**Published:** 2024-09-09

**Authors:** Yu Zhang, Shengjie Wang, Chaochao Zhang, Meng Qi, Luoqi Liu, Lipeng Yang, Na Lian

**Affiliations:** State Key Laboratory of Tree Genetics and Breeding, College of Biological Sciences and Technology, Beijing Forestry University, Beijing 100083, China; yuzhang@bjfu.edu.cn (Y.Z.); wangshengjiemail@126.com (S.W.); zhangchao99512@163.com (C.Z.); qimqmm@163.com (M.Q.); liuluoqi0510@163.com (L.L.); yanglipeng@bjfu.edu.cn (L.Y.)

**Keywords:** IQD, cold stress, microtubules, phase separation, *Malus domestica*

## Abstract

Microtubules undergo dynamic remodeling in response to diverse abiotic stress in plants. The plant-specific IQ67 DOMAIN (IQD) family proteins serve as microtubule-associated proteins, playing multifaceted roles in plant development and response to abiotic stress. However, the biological function of *IQD* genes in apple remains unclear. In this study, we conducted a comprehensive analysis of the *Malus domestica* genome, identifying 42 *IQD* genes distributed across 17 chromosomes and categorized them into four subgroups. Promoter analysis revealed the presence of stress-responsive elements. Subsequent expression analysis highlighted the significant upregulation of *MdIQD17* and *MdIQD28* in response to cold treatments, prompting their selection for further functional investigation. Subcellular localization studies confirmed the association of *MdIQD17* and *MdIQD28* with microtubules. Crucially, confocal microscopy and quantification revealed diminished microtubule depolymerization in cells transiently overexpressing *MdIQD17* and *MdIQD28* compared to wild-type cells during cold conditions. In conclusion, this study provides a comprehensive analysis of *IQD* genes in apple, elucidating their molecular mechanism in response to cold stress.

## 1. Introduction

The microtubule is a dynamic structure composed of α-tubulin and β-tubulin heterodimers [1]. Microtubule networks continually change, forming or dissolving in eukaryotic cells, which is essential for maintaining cellular integrity and function. Consequently, microtubules can physically and biochemically link cells to the external environment [2]. For instance, cold stress induces rapid microtubule depolymerization, followed by the assembly of new microtubule networks, which is crucial for initiating cold adaptation in plants [1,3,4]. Microtubule-associated proteins (MAPs) are proteins that regulate microtubule functions and dynamics [5]. These proteins interact with microtubules to modulate their stability, organization, and interaction with other cellular components. Numerous MAPs function as a sensory hub, decoding signals to modulate microtubule dynamics during plant development and environmental responses [2]. In *Arabidopsis*, PCaP2 destabilizes microtubules, potentially playing a role in chilling stress [6]. However, most MAPs, especially those in horticultural crops, remain functionally unexplored.

Cold stress significantly impacts many plants, affecting their growth, development, and geographical distribution, as well as crop quality and productivity [7,8,9]. The ability of plants to withstand cold stress is crucial for their survival. Plants have evolved intricate mechanisms to withstand cold stress at both physiological and molecular levels [10,11]. Cold signals are perceived by sensors, such as cellular membranes and calcium (Ca^2+^) channels [12,13]. Cold stress alters plasma membrane fluidity, which may be sensed by Ca^2+^ channels and other plasma membrane proteins, leading to increased [Ca^2+^]_cyt_ and activation of cold-responsive gene expression [13,14,15]. Subsequent studies have identified numerous components in cold stress signaling pathways, with the most well-characterized being the CBF-COR signaling pathway. *COR* (COLD REGULATED) genes are regulated by cold stress [16,17,18]. CBF (C-REPEAT BINDING FACTOR) directly binds to COR promoters, inducing their expression and enhancing plant freezing tolerance [19]. Additionally, research in alfalfa (*Medicago sativa*) cells has shown that drugs stabilizing the actin cytoskeleton inhibit *BN115*, a COR gene, while drugs destabilizing the actin cytoskeleton promote *BN115* expression [20]. These findings suggest that cytoskeleton remodeling plays a role in cold signal transduction.

The IQ67 DOMAIN (IQD) family is a group of plant-specific microtubule-associated proteins, characterized by the IQ domain containing 67 conserved amino acid residues, necessary for calmodulin interaction [21,22,23]. Recent research indicates that the DUF4005 domain of *Arabidopsis* IQD proteins can directly bind to microtubules and tubulin dimers [24]. Consequently, IQD proteins potentially bridge Ca^2+^ signaling and the microtubule cytoskeleton. For instance, IQD1 localizes to both the nucleus and the microtubules. IQD1 recruits kinesin light chain-related protein-1 (KLCR1) and CaM2 to microtubules in *Arabidopsis thaliana* [25]. IQDs regulate cell number and grain/fruit morphogenesis by altering microtubule structures in various plant species, such as *Arabidopsis*, rice (*Oryza sativa*), cucumber (*Cucumis sativus*), and watermelon (*Citrullus lanatus*) [26,27,28,29]. Additionally, IQDs play a role in different plant abiotic stress responses. In Chinese cabbage (*Brassica rapa ssp. pekinensis*), overexpressing *IQD5* enhances drought tolerance [30]. Conversely, in cotton (*Gossypium hirsutum*), reducing *GhIQD31* and *GhIQD32* expression increases susceptibility to drought and salt stress [31]. However, the regulatory mechanisms of IQDs in response to abiotic stress, particularly cold stress, remain unclear.

Apple (*Malus domestica*) is a vital economic fruit tree susceptible to cold-induced damage and death [32,33]. Understanding the regulatory mechanisms governing apple plant responses to cold is crucial for enhancing cold resistance germplasm. Prior research indicates the significant involvement of IQDs in various abiotic stress responses, yet knowledge of the apple IQD protein family remains limited. In this study, we identified 42 *IQDs* in the *Malus domestica* genome and conducted bioinformatic analyses. Additionally, we assessed their expression profiles in response to cold stress through gene expression analyses. Furthermore, we elucidated the roles of MdIQD17 and MdIQD28 in regulating microtubules under cold stress. These findings collectively underscore the functional significance of IQD family proteins in apple’s cold response regulation, shedding light on the associated microtubule regulatory mechanisms.

## 2. Results

### 2.1. Identification and Analysis of MdIQDs in the Apple Genome

**Figure 1:**
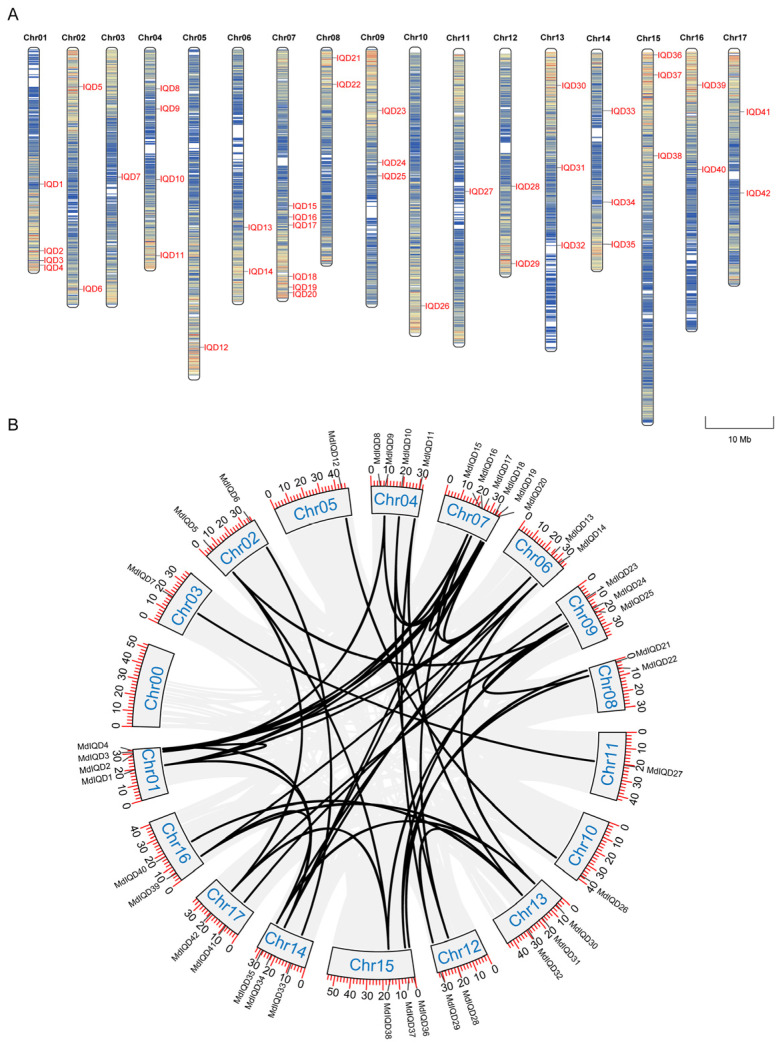
Chromosome location and synteny analysis of *MdIQDs*. A total of 42 *MdIQDs* were obtained in the apple genome. (**A**) Chromosome distribution analysis. (**B**) Synteny analysis. Duplicated *MdIQD* gene pairs were connected using the black lines.

To understand IQD proteins’ roles in apple, 42 *IQD* genes within *Malus domestica* were identified following candidate selection and verification, designated as *MdIQD1–MdIQD42* according to their chromosomal positions (from 1 to 17, top to bottom) (Table S1). Chromosome distribution analysis revealed uneven allocation of these 42 *MdIQD* genes across 17 *Malus domestica* chromosomes (Figure 1A), highlighting the diversification and complexity of the *IQD* family. Chromosome 7 (Chr07) harbored six genes, while Chr01 and Chr04 each contained four. Additionally, Chr09, Chr13, Chr14, and Chr15 featured three genes, while Chr02, Chr06, Chr08, Chr12, Chr16, and Chr17 hosted two genes each; the remaining chromosomes housed a single member. Gene duplication events during evolutionary processes were investigated using MCScanX, revealing the presence of homologous gene blocks on fewer chromosomes and the same chromosome (Figure 1B). Notably, Chr01 and Chr07 displayed blocks of homologous genes on the same chromosome, whereas Chr05 and Chr10, as well as Chr03 and Chr11, exhibited single pairs of homologous genes. These findings suggest that segmental duplication was essential in the emergence of the *MdIQD* gene family.

Detailed characteristics and information about these proteins are summarized in Table 1, encompassing amino acid count (AAs), molecular weight (MW), isoelectric point (pI), Instability Index (II), Aliphatic Index (AI), and Grand Average of Hydropathicity (GRAVY). As depicted in Table 1, MdIQD proteins ranged in size from 209 amino acids (MdIQD29) to 1001 amino acids (MdIQD21), with MW spanning from 23.28 KD (MdIQD29) to 109.77 KD (MdIQD21). With the exception of MdIQD21 and MdIQD36, the predicted isoelectric points exceeded seven, indicative of a prevalence of basic amino acids in MdIQD proteins. Furthermore, the subcellular localization was predicted based on the amino acid sequences of the proteins, and the results indicated that most of the proteins are localized to the nucleus (Table S2).

### 2.2. Evolutionary Analysis of MdIQDs

**Figure 2:**
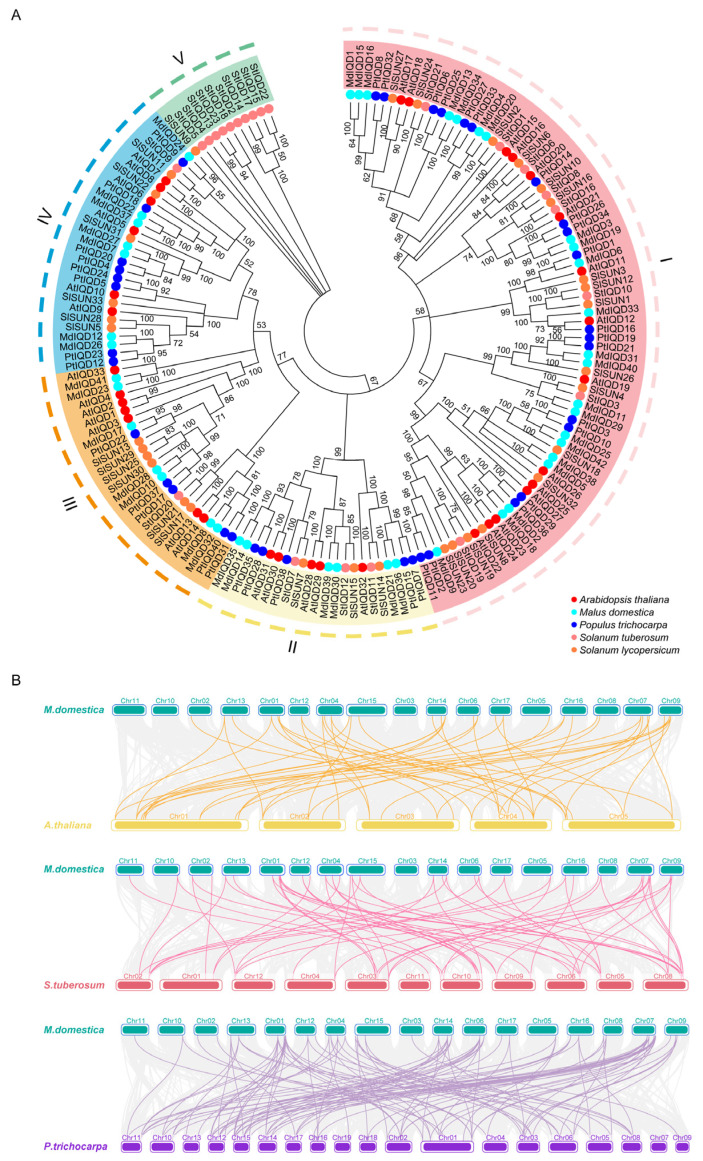
The phylogenetic tree and collinear relationship analysis between different genomes. (**A**) Phylogenetic analysis of the MdIQDs family. MdIQD family members highlighted by light blue round dot and other species distinguished by different colors and shapes. (**B**) Yellow line, pink line and purple line represent collinear gene pairs of *Malus domestica* and *Arabidopsis thaliana*, *Malus domestica* and *Solanum tuberosum*, *Malus domestica* and *Populus trichocarpa*, respectively.

To investigate the evolutionary relationships and potential functional characteristics of MdIQD family members, a phylogenetic tree was constructed using full-length IQD protein sequences from *Malus domestica*, *Populus trichocarpa*, *Arabidopsis thaliana*, *Solanum tuberosum*, and *Solanum lycopersicum*. The resulting phylogenetic tree included 42 MdIQDs, which were categorized into four subgroups (I, II, III, and IV) (Figure 2A). Subgroup I, comprising 22 members, was the largest, while Subgroup III and IV each contained seven MdIQD proteins. 

To analyze the homology and collinearity of *IQD* genes across different species, an interspecies collinearity analysis was performed using the multiple synteny plot function within TBtools v2.031 software. Comparing the collinear genomic blocks of *Malus domestica* with *Arabidopsis thaliana*, *Solanum tuberosum*, and *Populus trichocarpa* revealed homologous *IQD* genes due to chromosomal segmental duplication (Figure 2B). Notably, *Malus domestica* and *Populus trichocarpa* exhibited a higher number of collinear gene pairs, suggesting a closer evolutionary relationship between these two species.

### 2.3. Structural Characterization Analysis of MdIQDs

**Figure 3:**
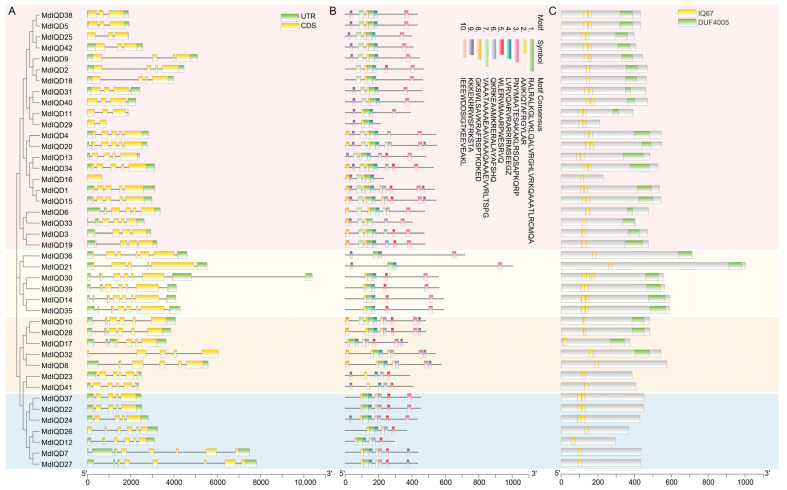
Phylogenetic relationship, gene structures, and conserved domains of MdIQDs. (**A**) The phylogenetic tree and gene structure of *MdIQD* genes. Green and yellow boxes represent UTR and CDS, respectively. (**B**) Motif compositions of MdIQD proteins. Different motifs highlighted with different colors. (**C**) Domain organization of MdIQD proteins.

To investigate the structure of *MdIQD* genes, the exon-intron structures were analyzed (Figure 3A). Varying numbers of introns were observed within the *MdIQD* gene family. Except for *MdIQD16* and *MdIQD29*, which possessed only one or two exons, the remaining *MdIQD* genes had a minimum of three exons, with most having five to six exons. The structure of MdIQD proteins was elucidated through the utilization of MEME Suite 5.5.7 software, which predicted ten conserved motifs that were designated as Motif 1 through Motif 10 (Figure 3B). The motif distribution analysis revealed that a well-conserved Motif 3 was present at the C-terminus of all members, while the N-terminal of most of these sequences contained well-conserved motifs (Motif 1, Motif 2, Motif 4, and Motif 7). All MdIQD proteins contain the IQ67 domain, and several members have the DUF4005 domain (Figure 3C). This common motif composition among most subgroup members implies potential similarities in their biological functions. 

**Figure 4:**
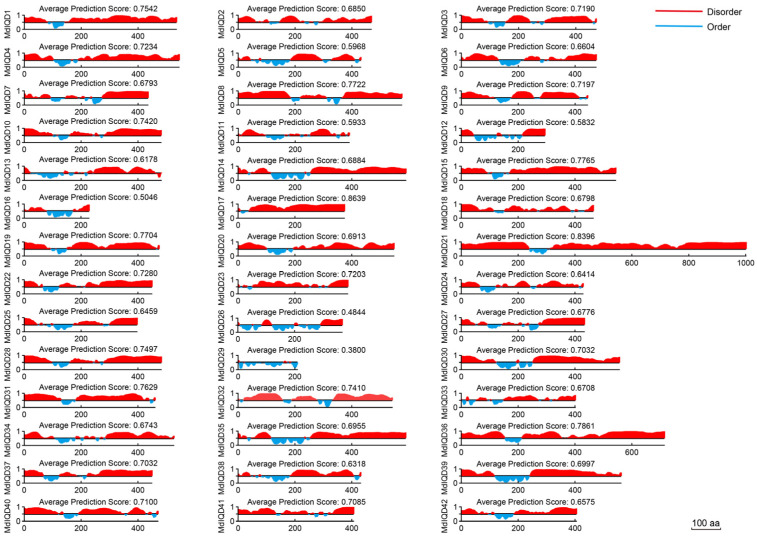
Intrinsically disordered regions prediction of 42 MdIQD proteins. The Predictor of Natural Disordered Regions (PONDR) website (http://www.pondr.com/) was utilized to project the intrinsically disordered regions (IDRs). A score exceeding 0.5 signifies a high degree of disorder within the sequence. The red part of the figure represents the disordered region of the protein.

Biological functions often involve phase separation, a ubiquitous phenomenon in cell biology. Phase separation is facilitated by intrinsically disordered regions (IDRs) within proteins. To assess the presence of IDRs in all MdIQDs, we employed the PONDR program designed for protein disordered region prediction (Figure 4). The analysis revealed that nearly all MdIQD proteins exhibited disordered regions, with prediction scores exceeding 0.5, except for MdIQD26 and MdIQD29. Notably, MdIQD17 and MdIQD21 displayed average prediction scores > 0.8. Furthermore, a significant proportion of MdIQDs displayed high disorder in their C-termini, underscoring the crucial role of the C-terminus in facilitating diverse biological functions through phase separation. 

### 2.4. Promoter Analysis and Expression Analysis under Cold Stress of MdIQDs

**Figure 5:**
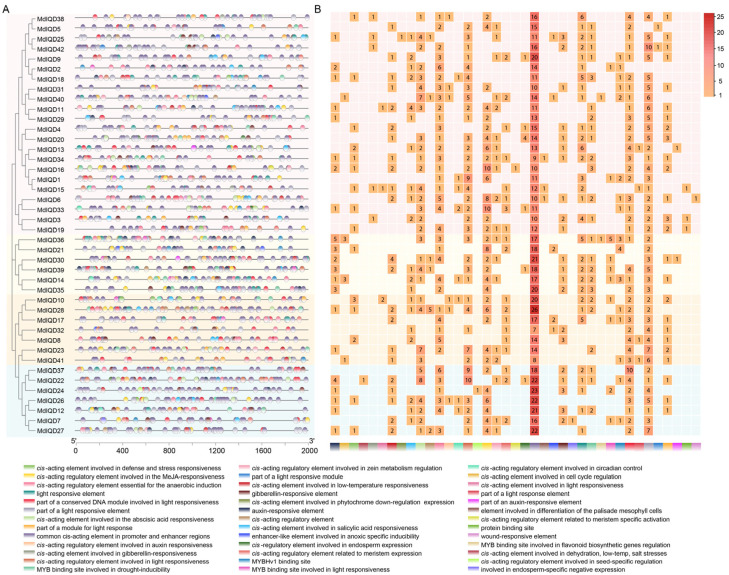
*Cis*-acting elements analysis of *MdIQD* promoters. (**A**) Distribution of *cis*-acting elements in *MdIQD* promoters. (**B**) Statistics of *cis*-acting elements in each promoter region. The heatmap demonstrated the number of *cis*-elements with the higher number in red color and lower number with orange color.

The potential roles of *MdIQD* genes in abiotic stress responses were explored by analyzing the *IQD* gene promoters along with 2000 bp genomic sequences upstream of the transcriptional start site using PlantCARE software (https://bioinformatics.psb.ugent.be/webtools/plantcare/html/, accessed on 1 August 2024) (Figure 5). The results revealed the presence of elements associated with hormone responses and environmental stimuli within *MdIQDs*. Hormone-responsive elements, such as abscisic acid, MeJA, and salicylic acid, were identified, alongside environmental stimuli elements including light-responsive elements, drought-inducibility, low-temperature responsiveness, and wound responsiveness. Notably, low-temperature responsiveness elements were found in 22 out of the 42 *IQD* family genes. These findings suggest a significant role of *MdIQDs* in plant responses to cold stress. 

**Figure 6:**
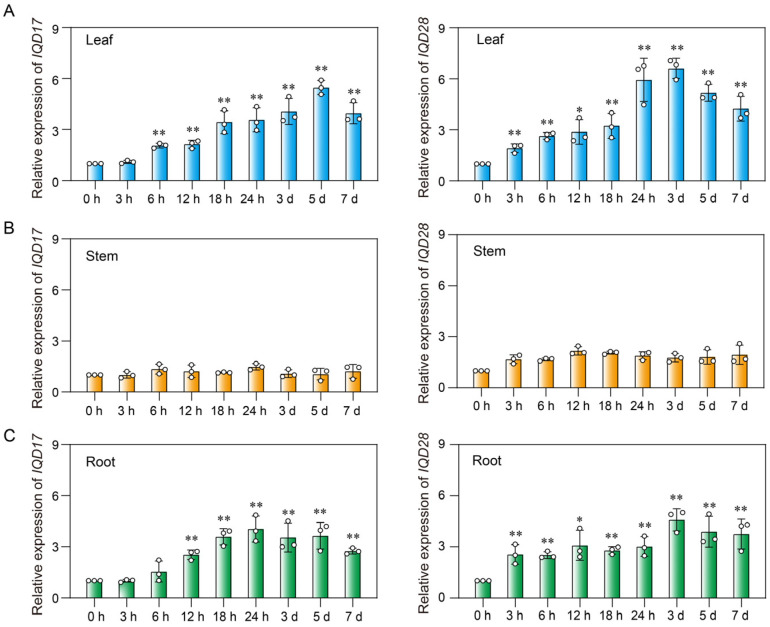
Expression profiles of *MdIQD17* and *MdIQD28* in different tissues under cold treatment. Three-month-old apple seedlings were treated at 0 °C for various durations, followed by the collection of leaves, stems, and roots for total RNA extraction and RT-qPCR analysis. (**A**) Relative expression of *MdIQD17* and *MdIQD28* in the leaves. (**B**) Relative expression of *MdIQD17* and *MdIQD28* in the stems. (**C**) Relative expression of *MdIQD17* and *MdIQD28* in the roots. *MdUBQ* was used as a reference gene. Error bars represent standard error of the mean (n = 3). * *p* < 0.05, ** *p* < 0.01.

To further dissect the involvement of *MdIQD* in plant cold stress responses, RNA-seq data from apple seedlings subjected to low-temperature stress were analyzed (Figure S1). *MdIQD* genes exhibited distinct expression patterns under cold stress compared to the control experiment. Specifically, *MdIQD17* and *MdIQD28* displayed heightened expression levels following cold stress in apple plants. Furthermore, qRT-PCR assays confirmed the increased expression of *MdIQD17* and *MdIQD28* in leaf and root tissues in response to cold stress, with no significant changes observed in stem tissues (Figure 6). Consequently, *MdIQD17* and *MdIQD28* were chosen for further functional studies. 

### 2.5. Subcellular Localization Analysis of MdIQD17 and MdIQD28

**Figure 7:**
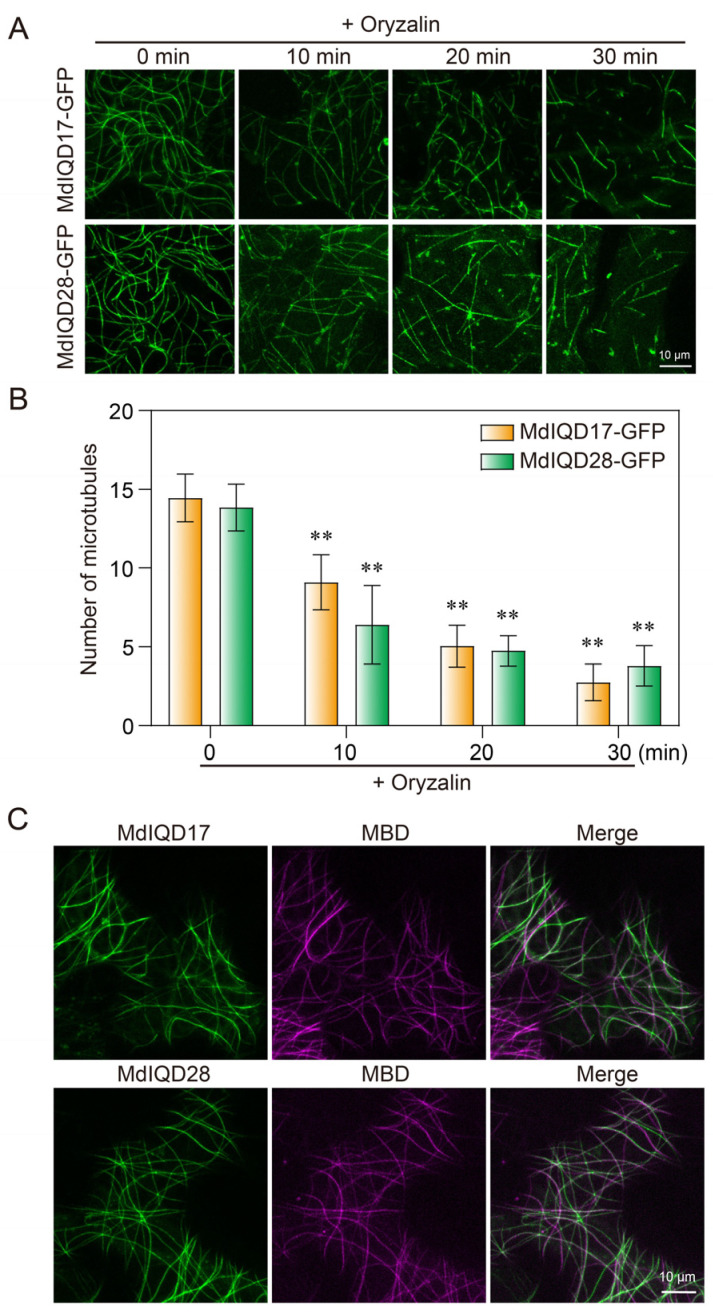
Subcellular localization of MdIQD17 and MdIQD28 proteins. (**A**) MdIQD17-GFP and MdIQD28-GFP transiently expressed in tobacco cells, respectively. The filamentous pattern of MdIQD17-GFP and MdIQD28-GFP were disrupted when the cells were treated with 10 μM oryzalin for 10 min, 20 min, and 30 min. (**B**) The graph shows the number of microtubules. ** *p* < 0.01. (**C**) Analysis of colocalization of transiently expressed MdIQD17-GFP and MdIQD28-GFP with MBD-mCherry.

Based on the above results, a significant cold stress response was observed in *MdIQD17* and *MdIQD28*. Therefore, *MdIQD17* and *MdIQD28* were cloned and investigated in the subsequent study. Constructs expressing MdIQD17-GFP and MdIQD28-GFP were generated and transiently introduced into tobacco leaf epidermal cells. Filamentous structures were observed in pavement cells using confocal microscopy. These filaments were disrupted by treatment with the microtubule-disrupting reagent oryzalin (Figure 7A,B). To validate this observation, co-expression of MdIQD17-GFP/MdIQD28-GFP and MBD-mCherry (labeling microtubules) was performed in tobacco leaf epidermal cells. Figure 7C demonstrates overlapping fluorescence between MdIQD17-GFP/MdIQD28-GFP and the red fluorescent signal of cortical microtubules, confirming the localization of MdIQD17 and MdIQD28 to microtubules. Considering the roles of *MdIQD17* and *MdIQD28* in the cold stress response, we assessed their subcellular localization in cells under normal growth conditions and in cells exposed to 0 °C for 2 h. We observed that MdIQD17-GFP and MdIQD28-GFP can undergo phase separation, partially forming punctate patterns induced by cold stress (Figure S2).

### 2.6. MdIQD17 and MdIQD28 Stabilize Microtubules under Cold Stress

**Figure 8:**
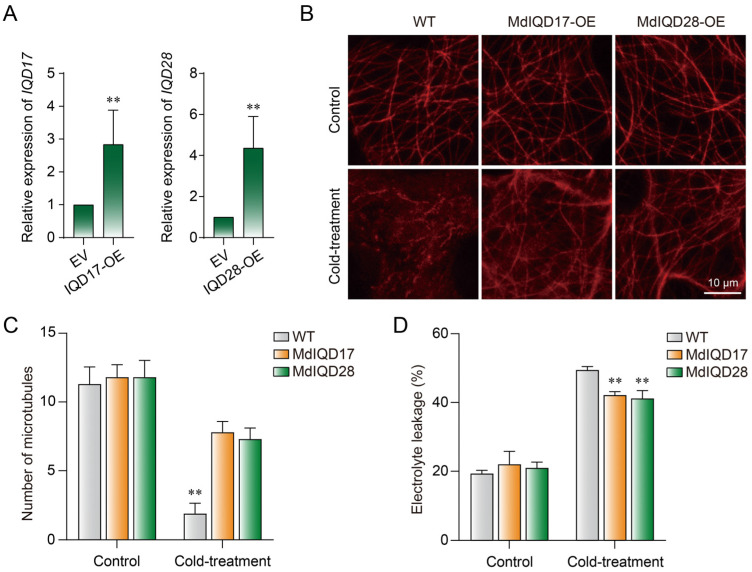
MdIQD17 and MdIQD28 enhanced microtubule stability in response to cold stress. (**A**) Relative expression level of *MdIQD17* and *MdIQD28* in tobacco leaves. EV, Empty Vector control. (**B**) Cortical microtubules in tobacco leaf cells from wild-type (WT), MdIQD17-OE, and MdIQD28-OE under cold treatment (0 °C, 2 h). Bar = 10 μm. (**C**) Quantification of cortical microtubules. ** *p* < 0.01 by *t*-test comparing the number of cortical microtubules in MdIQD17-OE and MdIQD28-OE cells with that in WT cells under the same conditions. (**D**) Electrolyte leakage from leaves of the tobacco leaves under cold treatment (0 °C, 2 h). ** *p* < 0.01.

MdIQD17 and MdIQD28 are localized to microtubules, which play a pivotal role in plant responses to cold stress. Hence, we postulated their involvement in regulating microtubule organization during cold stress. To examine this hypothesis, we assessed cortical microtubules in tobacco leaves transiently overexpressing *MdIQD17* and *MdIQD28* (Figure 8A). After two hours of cold exposure, microtubule density exhibited a slower decline in cells overexpressing *MdIQD17* and *MdIQD28* compared to control cells (Figure 8B). Specifically, microtubule density decreased by 83.2% in control cells, whereas in *MdIQD17* and *MdIQD28* overexpressed cells, the reductions were 33.9% and 38.1%, respectively (Figure 8C). The cold-induced electrolyte leakage of *MdIQD17* and *MdIQD28* overexpressed plants was also significantly lower than those of control plants before and after cold treatment, and no differences were observed under normal conditions (Figure 8D). These findings underscore the regulatory role of MdIQD17 and MdIQD28 in enhancing microtubule stability in response to cold stress. 

## 3. Discussion

IQDs, ubiquitous plant proteins found across land plants from mosses to vascular plants, play critical roles in plant development and environmental responses like cell shaping, basic host defenses, and drought resistance. Some members of the IQD protein family have been shown to be plant-specific MAPs. However, few IQD proteins have been characterized in apple plants.

IQD proteins contain the conserved IQ67 domain harboring one to three tandem repeats of the calmodulin-binding IQ motif containing isoleucine (I) and glutamine (Q) residues. Additionally, some IQD members possess the DUF4005 domain, indicating microtubule binding. In apple, 42 *IQD* genes were identified (Figure 1). Consistent with other reports, these genes were highly conserved, with most containing the DUF4005 domain (Figure 3). As per the phylogenetic tree, MdIQDs were divided into four subgroups. MdIQDs in Subgroup I and II contained the IQ67 and DUF4005 domains, except MdIQD16 and MdIQD29. MdIQDs in Subgroup IV lacked the DUF4005 domain, while AtIQD5 in Subgroup IV functions in microtubules, suggesting MdIQDs in Subgroup IV may also affect microtubules through unknown domains. Due to their diverse structures and subcellular localizations, different IQDs influence microtubule organization differently. AtIQD1 localizes to nuclei and microtubules, interacting with kinesin light chain-related protein-1 (KLCR1) and CaM, linking kinesin to Ca^2+^ second messenger signaling [25]. AtIQD13 associates with the plasma membrane and microtubules, regulating secondary cell wall pit formation in xylem cells [34]. AtIQD5 distributes evenly across cortical microtubules, regulating *Arabidopsis* leaf morphogenesis [28,35]. SlIQD21a physically interacts with SlMAP70s, regulating fruit elongation in tomatoes [36].

IQDs not only impact plant development and cellular morphogenesis but also enhance stress resistance in *Arabidopsis*, cabbage, corn, moso bamboo, and poplar [30,37,38,39]. AtIQD1 has been previously identified as a defense against aphids. ZmIQDs and PtIQDs respond to drought stress. The 26 *ZmIQD* genes in maize are regulated by drought stress, while *BrIQD5* shows potential as a target gene for improving cabbage’s drought tolerance. In this study, the promoters of *MdIQDs* contain numerous putative stress-related *cis* elements, suggesting their role in responding to environmental stress. Twenty-two *MdIQDs* exhibit responsiveness to low temperatures. The expression patterns of *MdIQDs* further confirm their involvement in cold stress response. Cold stress induces the expression of *MdIQD17* and *MdIQD28*, as demonstrated by the qRT-PCR analysis. To investigate MdIQDs’ function, we assessed cortical microtubules transiently overexpressing *MdIQD17* and *MdIQD28* in tobacco leaves. Overexpression of *MdIQD17* and *MdIQD28* stabilized microtubules under cold stress. These findings highlight the significant role of *MdIQD* genes in apple plants’ response to cold stress.

Cold stress is a major constraint for plants, and microtubules play a crucial role in their response to cold stress. In winter wheat, cold stress leads to rapid microtubule depolymerization, followed by the formation of new microtubule networks, which is essential for initiating cold adaptation [3]. Additionally, the drug taxol, which stabilizes microtubules, inhibits the development of freezing tolerance [40]. These findings highlight the significance of microtubule organization in plants under cold conditions. MAPs are involved in regulating this process. When exposed to cold stress, the MAP PCaP2 is highly induced to destabilize microtubules, thereby enhancing plant chilling tolerance [6]. However, the precise mechanisms governing biphasic microtubule reorganization still require further investigation. In this study, we discovered that MdIQD17 and MdIQD28 have biological functions in apple response to cold stress. To elucidate their roles in cold stress response, we introduced MdIQD17 and MdIQD28 into plants and assessed microtubule stability. The results demonstrated higher microtubule densities in cells overexpressing *MdIQD17* and *MdIQD28* compared to control cells under cold stress (Figure 8), suggesting that MdIQD17 and MdIQD28 participate in the cold stress response by influencing microtubule stability in apple. In conclusion, our findings establish MdIQD17 and MdIQD28 as important regulators in response to cold stresses.

## 4. Materials and Methods

### 4.1. Identification of IQDs in Malus domestica

To identify IQD proteins in *Malus domestica*, genome sequences of *Malus domestica* were obtained from the Phytozome database (https://phytozome-next.jgi.doe.gov/, accessed on 15 August 2023). *Malus domestica* IQD (MdIQD) proteins were searched using the BLASTP program, utilizing published *Arabidopsis thaliana* IQD protein sequences and their IQ67 domains as query sequences. Candidate sequences were confirmed by matching them to Pfam number PF00612 from the Pfam database (http://pfam.xfam.org/) and using the SMART program [41]. Sequences of 33 *Arabidopsis thaliana* IQD (AtIQD) proteins, 34 *Solanum lycopersicum* IQD (SlIQD/SlSUN) proteins, 34 *Populus trichocarpa* IQD (PtIQD) proteins, and 23 *Solanum tuberosum* IQD (StIQD) proteins were downloaded from Phytozome v13 (https://phytozome-next.jgi.doe.gov/, accessed on 20 August 2023).

### 4.2. Phylogenetic Analysis

The IQ67 domains of all MdIQD conserved domains were subjected to multiple sequence alignment and shading using DNAMAN8 software. ClustalW was employed to align these IQD sequences. Phylogenetic relationships of MdIQD proteins, along with those of *Arabidopsis thaliana*, *Solanum lycopersicum*, *Solanum tuberosum*, and *Populus trichocarpa*, were constructed using MEGA 7.0 software. Bootstrapping with 1000 replicates and pairwise deletion was performed. Orthologous pairs were identified following established criteria in previous reports [39].

### 4.3. Characteristic Analysis of Gene and Protein Sequences

The physicochemical parameters of each gene were generated using the ExPASy program (https://web.expasy.org/protparam/, accessed on 1 September 2023). The subcellular localization prediction for all proteins was performed using Cell-Ploc2.0 (A package of web-servers for predicting subcellular localization of proteins in different organisms, http://www.csbio.sjtu.edu.cn/bioinf/Cell-PLoc-2/, accessed on 8 September 2023) [42]. Conserved motifs in MdIQD were predicted via an online website. TBtools software was employed for integrated analysis, visualizing motif compositions, and protein structures [43]. The chromosomal distribution of *MdIQD* genes was confirmed and visualized using Mapchart 2.32 software based on Phytozome’s GFF information. Promoter sequences located 2000 bp upstream of *MdIQD* genes were extracted from Phytozome and analyzed for *cis*-acting elements using the PlantCare database [44].

### 4.4. Species Homology and Chromosome Localization

Gene chromosomal positions were mapped using TBtools software, utilizing annotated genomic structural information from *Arabidopsis thaliana*, *Populus trichocarpa*, *Solanum lycopersicum*, *Solanum tuberosum*, and *Malus domestica*, all obtained from Phytozome. Advanced circos functions in TBtools were applied to visualize data within the *Malus domestica* species. To visualize collinearity between *Malus domestica* and *Arabidopsis thaliana*, *Malus domestica* and *Populus trichocarpa*, and *Malus domestica* and *Solanum tuberosum*, the multiple synteny plot function in TBtools software was employed.

### 4.5. Intrinsically Disordered Regions Analysis

The online PONDR program predicted IDRs for all MdIQD proteins (http://www.pondr.com/). The presence of IDRs facilitates the formation of droplet shapes, inducing phase transition generation and regulation.

### 4.6. Analysis of Malus domestica RNA-seq under Cold Stress

The published transcriptome data (NCBI database Accession No: PRJCA013120) were utilized from cold-treated apples to analyze *MdIQDs* expression patterns [45]. Differentially expressed genes (DEGs) extracted from the RNA-seq data for all *IQD* family genes in apples were subjected to cold stress. The expression trends and interactions among DEGs over time were analyzed. FPKM values for *IQD* family genes were extracted at 8 h, 3 d, and 7 d post-cold treatment and represented as log_2_ (FPKM + 1). All *IQD* family genes were identified from the RNA-seq data of cold-stressed apple.

### 4.7. Plant Materials and Cold Treatments

Apple seedlings were cultivated in tissue culture within a growth chamber for a period of three months. Subsequently, tissue materials from the roots, stems, and leaves were employed for tissue-specific experiments and stress treatments. Apple variety selection and cultivation conditions were consistent with previous research [46]. Cold treatment was administered at 0 °C, while the control treatment was at 23 °C. Samples of leaves, stems, and roots were collected at 0 h, 3 h, 6 h, 12 h, 18 h, 24 h, 3 d, 5 d, and 7 d post-treatment, with each sample having three biological replicates. The control group remained untreated, while the experimental group received the same conditions as the control group except for the stress treatment. All materials were flash-frozen in liquid nitrogen and stored at −80 °C.

### 4.8. Total RNA Extraction and cDNA Reverse Transcription Synthesis

Total RNA was extracted using the Eastep Super Total RNA Extraction Kit (LS1040, Shanghai Promega, Shanghai, China), followed by quality assessment using the NanoDrop 8000 (Thermo Fisher Scientific, Waltham, MA, USA). Subsequently, 1 µg of RNA was reverse transcribed into cDNA using the rapid quantitative RT Supermix Kit and stored at −20 °C for qRT-PCR experiments.

### 4.9. Quantitative Real-Time PCR (qRT-PCR)

Specific qRT-PCR primers were designed with Primer 3 plus software (https://www.primer3plus.com/index.html, accessed on 25 September 2023), selecting the most specific primers with a length of ~20 bp and a sequence range of ~200 bp (Table S3). UBQ served as the reference gene for expression data normalization. A qRT-PCR was conducted on a CFX connect system with a 40 µL per well setup. Data analysis employed the 2^−ΔΔCt^ method along with a Student’s *t*-test for significance assessment. Each sample comprised three biological replicates, and each biological replicate underwent three technical replicates.

### 4.10. Subcellular Localization of MdIQD17 and MdIQD28 Proteins

Firstly, we extracted RNA from apple using the CTAB method and then reverse transcribed the RNA to obtain cDNA using the HiFiScript gDNA Removal cDNA Synthesis Kit (CW2582M) (CoWin Biosciences, Taizhou, Jiangsu Province, China). Subsequently, we cloned the sequences of the CDS regions of *MdIQD17* and *MdIQD28* from the apple cDNA. The sequences of *MdIQD17*, *MdIQD28* and *GFP* were amplified and inserted into the pCAMBIA1390 vector to generate the *35S*:*MdIQD17-GFP* and *35S*:*MdIQD28-GFP* plasmids. The sequences of *MBD* (microtubule binding domain of MAP4) and *mCherry* were amplified and inserted into the pCAMBIA1390 vector to generate the *35S:MBD-mCherry* plasmid. The resulting plasmid was transformed into *Agrobacterium tumefaciens* GV3101 and infected tobacco leaves. Subcellular localization of MBD-mCherry, MdIQD17-GFP, and MdIQD28-GFP were visualized in tobacco leaves using a confocal laser scanning microscope system Zeiss LSM 880 (Carl Zeiss AG, Oberkochen, German) [47,48]. 

For the oryzalin treatment, tobacco leaves were subjected to a 10 μM oryzalin solution for durations of 10, 20, and 30 min. In the case of cold treatment, the leaves were exposed to a temperature of 0 °C for a period of 2 h.

### 4.11. Quantification of Cortical Microtubules

As previously described by Li et al. [49], we used ImageJ2 software to assess the density of cortical microtubules within the cells. A vertical line was delineated perpendicular to the predominant orientation of the cortical microtubules, and the crossing density of these microtubules was measured. Each cell was subjected to this measurement three times, and at least 10 cells were analyzed per treatment group. The values were recorded, and significant differences were analyzed using a paired Student’s *t*-test.

### 4.12. Electrolyte Leakage Analysis

According to the previous method of measuring electrolyte leakage [50], leaf samples were obtained by creating 1 cm diameter discs from plant leaves post-cold stress treatment. These discs were submerged in deionized water, subjected to a vacuum for 5 min to ensure full immersion, and then allowed to stand for 30 min to equilibrate. The extent of electrolyte leakage was determined by calculating the ratio S1/S2. Here, S1 denotes the initial conductivity reading taken with a conductance meter (model DDS-307A, REX, Shanghai, China), and S2 represents the final conductivity value, which was recorded after the leaf discs were boiled for 10 min to release all electrolytes.

## 5. Conclusions

A comprehensive analysis of 42 IQD family members in *Malus domestica* revealed their highly conserved nature. Promoter sequence analysis identified *cis*-acting elements related to cold stress. qRT-PCR data mining showed significant responsiveness of *MdIQD17* and *MdIQD28* to cold stress. Additionally, MdIQD17 and MdIQD28 were found to co-localize and stabilize with cortical microtubules in tobacco cells. Furthermore, the regulatory roles of MdIQD17 and MdIQD28 in enhancing microtubule stability in response to cold stress were confirmed. Therefore, these findings provide direct evidence of the functional roles of MdIQD proteins in cold stress response and lay the groundwork for future studies on IQD genes’ involvement in stress tolerance.

## Figures and Tables

**Table 1:** Physical and chemical characteristics of MdIQD proteins.

Protein	Number of Amino Acid	Molecular Weight (Da)	Theoretical pI	Instability Index (%)	Aliphatic Index (%)	Grand Average of Hydropathicity (GRAVY)
MdIQD1	535	60,091	10.41	69.61	55.91	−0.95
MdIQD2	468	51,603	10.50	55.29	58.93	−0.75
MdIQD3	471	52,989	9.77	60.18	54.12	−0.97
MdIQD4	544	61,121	10.08	66.68	58.20	−0.90
MdIQD5	431	47,034	10.24	50.69	64.20	−0.54
MdIQD6	474	53,895	10.00	74.35	65.74	−0.76
MdIQD7	435	48,357	10.07	56.64	68.28	−0.83
MdIQD8	575	64,387	10.94	65.22	50.12	−0.96
MdIQD9	444	49,035	10.29	60.41	59.71	−0.75
MdIQD10	481	53,551	10.18	63.79	66.76	−0.78
MdIQD11	390	44,228	10.43	47.08	67.82	−0.84
MdIQD12	294	32,996	10.09	52.54	67.11	−0.72
MdIQD13	482	54,186	10.07	56.89	64.09	−0.78
MdIQD14	590	64,470	9.85	50.44	75.03	−0.67
MdIQD15	543	61,108	10.40	72.33	56.19	−0.97
MdIQD16	228	26,093	10.19	45.06	71.58	−0.58
MdIQD17	374	41,714	11.17	78.12	49.17	−0.99
MdIQD18	464	51,454	10.48	62.02	57.31	−0.74
MdIQD19	474	52,600	9.66	58.47	53.00	−0.97
MdIQD20	548	61,162	10.17	65.85	58.14	−0.87
MdIQD21	1001	109,765	4.91	67.91	59.13	-0.97
MdIQD22	449	50,382	10.27	49.23	55.90	−0.84
MdIQD23	386	43,424	9.33	60.49	49.25	-0.94
MdIQD24	428	48,167	10.19	50.31	60.40	−0.82
MdIQD25	397	44,785	9.93	65.38	57.86	−0.75
MdIQD26	367	41,220	10.34	50.60	79.05	−0.46
MdIQD27	433	47,871	10.07	56.05	68.15	−0.85
MdIQD28	482	53,797	10.09	63.76	63.80	−0.84
MdIQD29	209	23,277	11.32	38.06	95.31	−0.19
MdIQD30	556	60,935	9.92	54.73	75.52	−0.68
MdIQD31	460	50,918	9.82	57.21	54.89	−0.79
MdIQD32	542	61,072	10.41	64.54	53.87	−0.94
MdIQD33	401	45,295	10.14	51.33	71.37	−0.71
MdIQD34	526	58,926	9.93	64.54	61.16	−0.83
MdIQD35	589	64,370	9.85	49.50	72.85	−0.72
MdIQD36	714	77,924	5.57	65.74	64.19	−0.88
MdIQD37	449	50,343	10.22	51.41	57.84	−0.85
MdIQD38	431	47,428	10.27	52.09	61.25	−0.67
MdIQD39	562	61,994	9.76	50.42	72.31	−0.73
MdIQD40	470	52,206	9.88	59.97	56.64	−0.79
MdIQD41	406	45,574	9.39	67.43	55.47	−0.87
MdIQD42	405	45,263	10.26	59.47	58.42	−0.69

## Data Availability

The raw data supporting the conclusions of this article will be available from the authors upon request.

## Supplementary Materials

The following supporting information can be downloaded at: https://www.mdpi.com/article/10.3390/plants13172532/s1. Table S1: List of gene name. Table S2: List of subcellular localization predictions. Table S3: List of primers. Figure S1: Heat map was used to analyze the expression level of *MdIQD* family genes under cold treatment. Figure S2: Subcellular localization of MdIQD17 and MdIQD28 proteins after treatment with 0 °C for 2 h.
