# Supplementary figures and images for: Genome-Wide Characterization of IQD Family Proteins in Apple and Functional Analysis of the Microtubule-Regulating Abilities of MdIQD17 and MdIQD28 under Cold Stress
Source: Plants (Basel). 2024 Sep 9;13(17):2532. doi: 10.3390/plants13172532 (PMC11397337; doi:10.3390/plants13172532)

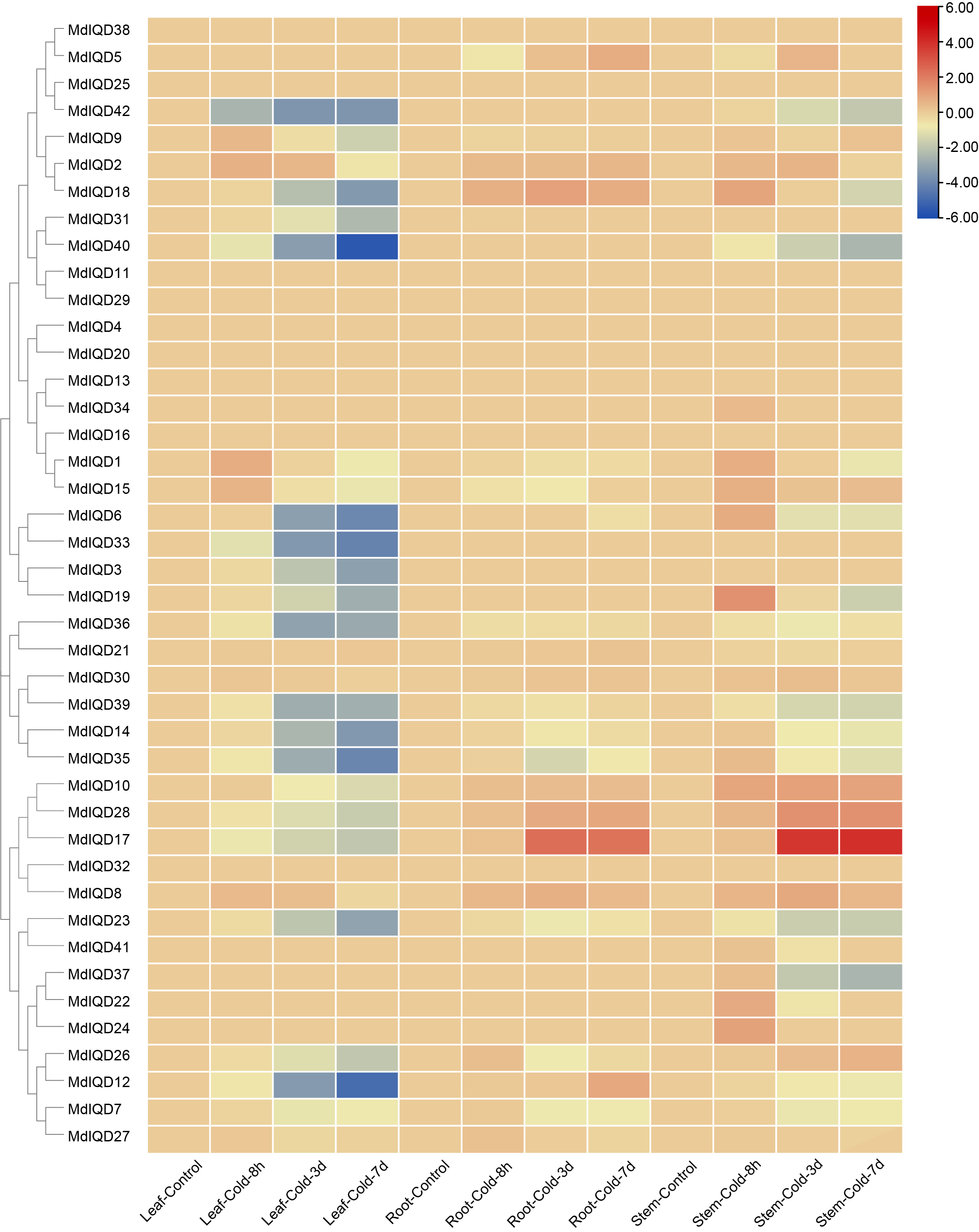

Supplement: Supplementary file 1 [file plants-13-02532-s001.zip › Fig. S1.jpg]

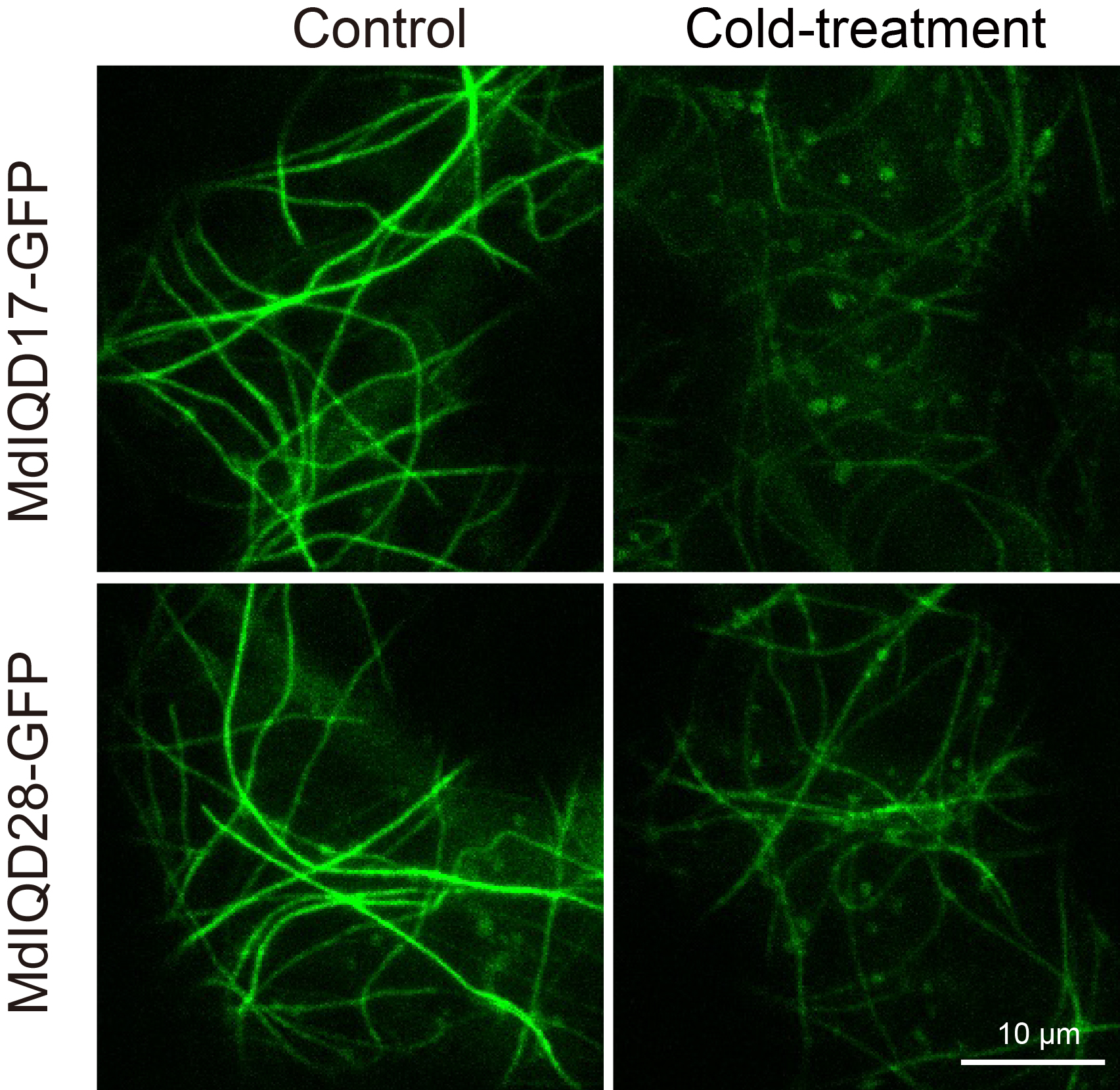

Supplement: Supplementary file 1 [file plants-13-02532-s001.zip › Fig. S2.jpg]
